# Supplementary material for: DREAM: A Toolbox to Decode Rhythms of the Brain System
Source: Neuroinformatics. 2021 Jan 7;19(3):529–45. doi: 10.1007/s12021-020-09500-9 (PMC8233299; doi:10.1007/s12021-020-09500-9)
Supplement: Supplementary file 1 — (PDF 176 MB) [file 12021_2020_9500_MOESM1_ESM.pdf]

## Appendix: Pseudo-codes of Core Algorithms

**Algorithm 1:** Frequency Decomposition, O($T_R$) complexity

**Input:** double $T_R$: sampling rate (seconds), int $N$: number of time points ;

**Output:** array $fbands$: an array of all the decomposed frequency bands;

```
/* determine the detectable frequencies */
fmax = 1/(2 * T_R);
fmin = 1/(N * T_R/2);
j = 0;
fnum = ceil(N/2)
for (i = 0; i < fmax, i++) : do
    freq[i] = fmax/(fnum + 1) * i;
    if (freq[i] < fmin) then
        idx[j] = i;
        j++;
    end
    frmin = { freq(idx(j − 4))   j > 4,
            { freq(idx(j))/2     else.
end
/* determine the usable frequency bands with the N3L theory */
nlcfmin = round(log(frmin));
nlcfmax = round(log(fmax));
for (i = nlcfmin; i < nlcfmax; i++) : do
    nlfc[i] = i;
end
for (i = 1; i < max(nlfc); i++) : do
    idxfmin = min(abs(freq − e^(nlcf(N)−0.5)));
    idxfmax = min(abs(freq − e^(nlcf(N)+0.5)));
    fbands[i, 1] = freq(idxfmin);
    fbands[i, 2] = freq(idxfmax)];
end
/* modify the min frequency band and max frequency band */
tmpf = fbands[1];
if tmpf(1) < frmin then
    tmpf(1) = frmin;
    fbands[1] = tmpf;
end
tmpf = fbands{end};
if tmpf(2) > fmax then
    tmpf(2) = fmax;
    fbands{end} = tmpf;
end
```

**Algorithm 2:** Bandpass Filtering, O($N \times T \times \ln N$) complexity

**Data:** array *input_mtx* : a $T \times N$ data matrix, where $N$ is the number of time points, $T$ is the number of time series. $input\_mtx(t, n)$ denotes the signal intensity of the $t^{\text{th}}$ time series in the $n^{\text{th}}$ time point.

**Input:** double $low\_f, high\_f, T_R$

**Output:** array *out_mtx* : a $T \times N$ matrix, denoting the bandpass filtered matrix

int $f = 0$;

double $fs = 1/T_R$;

**if** $N\ MOD\ 2 = 0$ **then**

$$f[j] = \begin{cases} j & j = 1 : N/2, \\ N - j & j = N/2 : N. \end{cases}$$

**else**

$$f[j] = \begin{cases} j & j = 1 : (N-1)/2 \\ N - j & j = N/2 : N. \end{cases}$$

**end**

`/* create a rectangle window according to the cutoff frequency */`

**for** $(i = 0; i < N; i++)$ : **do**

$$ind[i] = \begin{cases} i & (low\_f <= f \&\& f <= high\_f), \\ 0 & \text{else.} \end{cases}$$

$$rectangle[i] = \begin{cases} 1 & ind[i] = 1, \\ 0 & \text{else.} \end{cases}$$

**end**

`/* use fft to transform the time series into frequency domain */`

$mtx\_fft = fft(input\_mtx)$ ;

`/* ifft with the rectangle to convert data back to time domain */`

$output\_mtx = ifft(mtx\_fft, rectangle)$
